# Interleukin-6, tumour necrosis factor α and interleukin-1β in patients with renal cell carcinoma

**DOI:** 10.1038/sj.bjc.6600257

**Published:** 2002-05-06

**Authors:** N Yoshida, S Ikemoto, K Narita, K Sugimura, S Wada, R Yasumoto, T Kishimoto, T Nakatani

**Affiliations:** Department of Urology, Osaka City University Medical School 1-4-3 Asahi-machi, Abeno-ku, Osaka 545-8585, Japan

**Keywords:** IL-6, TNFα, IL-1β, renal cell carcinoma

## Abstract

As regulators of malignant cell behaviour and communication with stroma, cytokines have proved useful in understanding cancer biology and developing novel therapies. In renal cell carcinoma, patients with inflammatory reactions are known to have poor prognosis. In order to elucidate the relation between renal cell carcinoma and the host, serum levels of inflammatory cytokines, interleukin-6, tumour necrosis factor α, interleukin-1β, were measured. One hundred and twenty-two patients with renal cell carcinoma and 21 healthy control subjects were studied, and serum cytokine levels were measured using a highly sensitive ELISA kit. As a result, in the control group, interleukin-6, tumour necrosis factor α and interleukin-1β levels were 1.79±2.03, 2.74±0.94 and 0.16±0.17 pg ml^−1^, respectively. In the renal cell carcinoma patients, they were 8.91±13.12, 8.44±4.15 and 0.53±0.57 pg ml^−1^, respectively, and significantly higher. In the comparison of stage, interleukin-6 level was significantly higher in the stage IV group compared to the other stage groups including the control group, while tumour necrosis factor α level was significantly higher in each stage group compared to the control group. As for grade, interleukin-6 level was significantly higher in the grade 3 group compared to the control, grade 1 and grade 2 groups, while tumour necrosis factor α level was significantly higher in each grade group compared to the control group. All cytokines had a positive correlation with tumour size. In regard to the correlation with CRP, all cytokines had a positive correlation with CRP, while interleukin-6 had a particularly strong correlation. In conclusion, interleukin-6 may be one of the factors for the poor prognosis of patients with renal cell carcinoma. In addition, tumour necrosis factor α may be useful in the early diagnosis of renal cell carcinoma and post-operative follow-up.

*British Journal of Cancer* (2002) **86**, 1396–1400. DOI: 10.1038/sj/bjc/6600257 www.bjcancer.com

© 2002 Cancer Research UK

## 

Renal cell carcinoma (RCC) is the most common malignant tumour of the kidney. As regulators of malignant cell behaviour and interaction with the host, cytokines have proved useful in understanding cancer biology and developing novel therapies. Among these cytokines, interleukin-6 (IL-6) has been frequently studied in renal cell carcinoma (RCC). Miki *et al* (1989) reported that freshly isolated RCC tissues expressed mRNA of IL-6 and secreted active IL-6, and that IL-6 antiserum specifically inhibited *in vitro* tumour growth. Takenawa *et al* (1991) reported that primary RCC tissues and RCC cell lines expressed mRNA of IL-6 and IL-6 receptors, and that patients with a high level expression of IL-6 had significantly greater incidences of lymph node metastasis and a larger increase in serum c-reactive protein (CRP) than those without it. In addition, fresh RCC cells and cell lines have been reported to produce IL-6 (Blay *et al*, 1994; Chang *et al*, 1997). These results suggest that IL-6 acts as an autocrine growth factor for RCC cells. Blay *et al* (1992) reported that serum IL-6 was detectable in 66 of 138 patients (48%), and that serum CRP correlated with IL-6 levels. The interval between diagnosis of the primary tumour and metastasis was shorter in patients with a detectable serum IL-6 and/or serum CRP level >50 mg l^−1^, and they concluded that serum IL-6 and CRP levels were adverse prognosis factors in patients with metastatic renal cell carcinoma. They also reported that the presence of high pretreatment serum IL-6 levels inversely correlated to the response to IL-2 and to survival after immunotherapy with IL-2 in patients with RCC (Blay *et al*, 1990). Tsukamoto *et al* (1992) reported that serum IL-6 was detected in 18 out of 71 patients (25%). They indicated that although IL-6 level did not directly correlate with tumour volume and differentiation grade of the carcinoma, the positive rate increased with progression of the stage, and that serum IL-6 level affected the 5-year survival of patients without distant metastasis. Furthermore, Stadler *et al* (1992) and Dosquet *et al* (1994) reported that serum IL-6 was a good marker for metastases in RCC patients. However, as these studies did not use a high sensitivity method, the obtained values were lower than the minimum detectable levels in many of the patients. In this study, we used a highly sensitive ELISA kit in order to study in more detail the significance of serum IL-6 in RCC patients.

Tumour necrosis factor α (TNFα) and interleukin-1β (IL-1β) are pleiotropic mediators of biologic responses related to infection, immunity, and inflammation (Le and Vilcek, 1987). IL-1, TNFα and IL-6 are cytokines with overlapping biological properties which form a complex network of interactive signals. They are considered major mediators of fever and the production of acute phase proteins (Baumann and Gauldie, 1990). There have been few reports on serum TNF and serum IL-1 in RCC. In multivariate analysis for proportional hazard regression, Dosquet *et al* (1997) reported that TNFα was an independent prognostic indicator, with a normal plasma TNFα being highly predictive for a good prognosis in patients with untreated renal cell carcinoma. Determining the levels of these cytokines in the serum may not only help in understanding the biology of the tumour, but they may also be useful as a diagnosis. In this study, we measured the serum levels of the cytokines in RCC patients using a highly sensitive ELISA kit and also investigated these cytokines in the supernatant of short-term and long-term established RCC cells.

## MATERIALS AND METHODS

### Patients

One hundred and twenty-two patients with RCC (mean age: 62.4 years) were studied. Histological examination of their tumours indicated RCC in all cases. Tumour stages were classified according to Robson's classification. The patients were also divided into three groups according to grade. They did not receive any treatment before the admission to the study. Twenty-one healthy control subjects (mean age : 61.9 years) were also studied.

### Assay of IL-6, TNFα and IL-1β in serum

Serum from the peripheral venous blood of the patients and healthy control subjects was frozen at −80°C until use. Serum cytokine levels were measured using a highly sensitive ELISA kit (Genzyme Techne Analyza Immunoassay System human IL-6, TNFα, IL-1β High Sensitivity). The minimum detectable concentrations were estimated to be 0.094 pg ml^−1^ for IL-6, 0.18 pg ml^−1^ for TNFα and 0.1 pg ml^−1^ for IL-1β.

### Short-term and long-term established RCC lines

Tumour cells were isolated by enzymatic digestion of surgically removed tumour tissues. Short-term cultured RCC were established by culturing the tumour cell suspension in a 24-well plastic tissue culture cluster (Corning Inc., NY, USA) in RPMI 1640 (Nikken Bio Medical Laboratory, Japan) supplemented with 10% foetal calf serum (FCS) (Equitech-BIO, Inc., Ingram, TX, USA). Cells were grown for 2 weeks and then split by the use of tripsin. Tumour cells that had been through 4 to 5 passages were characterised by immunostaining with antibodies to RCC (Monosen, San Francisco, CA, USA) and Uro2 (Signet, Dedham, MA, USA). In some of the patients, long-term cell culture of 20 passages or more were possible.

### Assay of IL-6, TNFα and IL-1β in cell culture supernatant

RCC lines (1×10^5^ cells ml^−1^) were cultured in RPMI 1640 supplemented with 10% FCS for 48 h. The supernatant was centifuged, passed through a Millipore filter (0.45 μ) and stored at −80°C until testing. Cytokine levels were measured using an ELISA kit (Genzyme Techne Analyza Immunoassay System human IL-6, TNFα, IL-1β). The minimum detectable concentrations were estimated to be 0.7 pg ml^−1^ for IL-6, 4.4 pg ml^−1^ for TNFα and 1.0 pg ml^−1^ for IL-1β.

### Statistical analysis

The data are presented as mean±standard deviation. The data were analysed by multiple comparison (Fisher's PLSD) after one-factor ANOVA. Regression lines were fitted by the method of least squares. Receiver operating characteristic (ROC) curves were constructed for IL-6, TNFα and IL-1β by calculating their sensitivity and specificity in differentiating RCC patients from healthy control subjects at several cutoffs.

## RESULTS

Serum levels of IL-6, TNFα and IL-1β in the RCC patients and healthy control subjects are summarised in Table 1

**Table 1:**
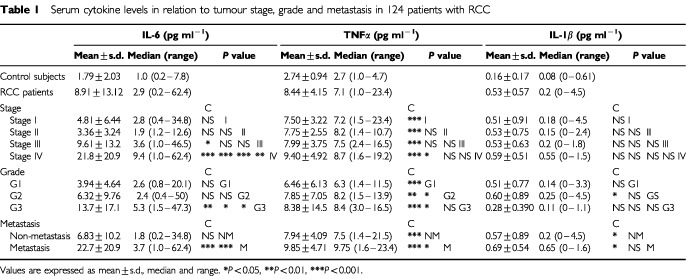
Serum cytokine levels in relation to tumour stage, grade and metastasis in 124 patients with RCC

. In the control subjects, IL-6 level was 1.79±2.03 pg ml^−1^, TNFα level was 2.74±0.94 pg ml^−1^ and IL-1β level was 0.16±0.17 pg ml^−1^, while in the RCC patients, IL-6 level was 8.91±13.12 pg ml^−1^, TNFα level was 8.44±4.15 pg ml^−1^ and IL-1β level was 0.53±0.57 pg ml^−1^ and significantly higher (IL-6: *P*<0.05, TNFα: *P*<0.0001, IL-1β, *P*<0.05). RCC patients were also divided according to stage (I, II, III, IV), grade (G1, G2, G3) and with or without lymph node or distal metastasis (metastasis, non-metastasis). When compared according to stage, IL-6 level was significantly higher in the stage IV group compared to the other stage groups including the control group, while TNFα level was significantly higher in each stage group compared to the control group. There was no significant difference in IL-1β level in among the groups. When compared according to grade, IL-6 level was significantly higher in the G3 group compared to the control, G1 and G2 groups, while TNFα level was significantly higher in each grade group compared to the control group. IL-1β level was significantly higher in the G2 group compared to the control group. In the comparison of with or without metastasis, IL-6, TNFα and IL-1β levels were significantly higher in the metastasis group compared to the control and non-metastasis groups. TNFα level was also significantly higher in the non-metastasis group compared to the control group (Table 1).

**Figure 1:**
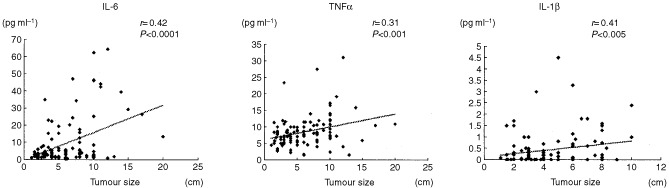
Correlation between serum concentrations of cytokines and tumour size in patients with RCC.

**Figure 2:**
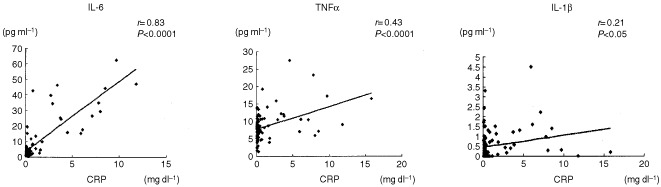
Correlation between serum concentrations of cytokines and CRP in patients with RCC.

There was a positive correlation between tumour size and IL-6, TNFα and IL-1β levels (IL-6: *r*=0.42, *P*<0.0001; TNFα: *r*=0.31, *P*<0.001; IL-1β: *r*=0.41, *P*<0.005) (Figure 1

). There was also a positive correlation between CRP and IL-6 and TNFα levels (IL-6: *r*=0.83, *P*<0.0001; TNFα: *r*=0.43, *P*<0.0001) (Figure 2

).

**Figure 3:**
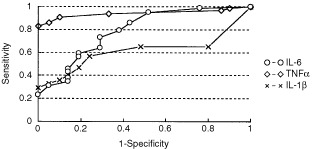
ROC curve of cytokines in healthy control subjects and patients with RCC.

The ROC curve analysis was used for the comparison of cytokine levels in the evaluation of RCC diagnosis. In this analysis, area under the curve (AUC) of serum TNFα was best compared to the other cytokines (Figure 3

).

Cytokine levels of the culture supernatants of short-term and long-term established RCC lines are shown in Table 2

**Table 2:**
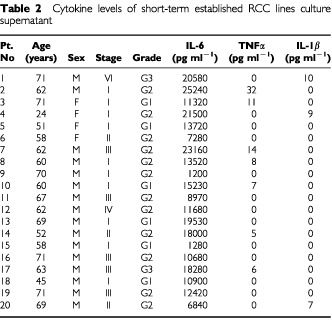
Cytokine levels of short-term established RCC lines culture supernatant

and Table 3

**Table 3:**
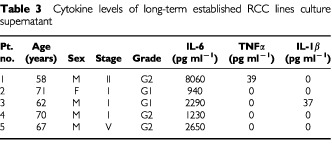
Cytokine levels of long-term established RCC lines culture supernatant

3. IL-6 level was high in all patients, and while TNFα and IL-1β levels could be detected in some of the cells, these levels were low in all cases and under minimum detectable concentrations in most cases. IL-6 tended to be lower in the long-term established RCC lines compared to the short-term established RCC lines.

## DISCUSSION

IL-6 is a pleiotropic cytokine secreted by a wide variety of cell types including lymphocytes, monocytes and tumour cells (Le and Vilcek, 1989; Kishimoto *et al*, 1992). There have been several reports that RCC can produce IL-6 and act as an autocrine growth factor (Blay *et al*, 1994; Chang *et al*, 1997). In our study of RCC using short-term and long-term established cell culture, IL-6 was also produced at high levels. These observations support the hypothesis that IL-6 may be a marker of tumour aggressiveness. Blay *et al* (1992) reported that serum IL-6 was positive in 48% of patients with RCC. Tsukamoto *et al* (1992) reported that it was positive in 25% and Hamao *et al* (1994) reported that it was positive in 53%. In our study, serum IL-6 could be detected in all patients in the control group and RCC group by using a highly sensitive ELISA kit in our present study. Tsukamoto *et al* (1992) and Hamao *et al* (1994) reported that the number of serum IL-6 positive patients increased with stage, and Dosquet *et al* (1994) reported the IL-6 blood levels were higher in patients with lymph node invasion and/or distant metastases. In our study, serum IL-6 level was significantly higher in the stage IV group compared to the control, stage I, stage II and stage III groups. Tsukamoto *et al* (1992) reported that there was no correlation of serum IL-6 with grade and tumour size, while Dosquet *et al* (1994) also reported that there was no correlation with tumour size. In another report, a correlation between serum IL-6 and tumour size was indicated in ovarian cancer (Berek *et al*, 1991). In our study, IL-6 was significantly higher in the grade 3 group compared to the grade 1 and grade 2 groups, and there was a positive correlation with tumour size. These results can be attributed to our use of the high sensitivity method. IL-6 exerts pyrogenic activity and induces the production of acute-phase proteins including CRP and haptoglobulin by liver cells *in vitro*. Blay *et al* (1992) and Dosquet *et al* (1994) reported that there was a positive correlation between serum IL-6 and CRP, we similarly found a positive correlation between serum IL-6, TNFα and IL-1β and CRP, but IL-6 had the strongest correlation.

TNFα is recognised as a potentially powerful immune mediator and multifunctional cytokine that regulates the central aspects of host defence responses and plays a major role in the pathogenesis of various immune disorders such as fever, septic shock, arthritis and inflammatory reaction (Brenner, 1988; Frei and Spriggs, 1989). IL-1β is involved in many host reactions including immunologic, inflammatory, endocrinologic and haemopoietic ones. Furthermore, IL-1β performs a synergistic role in potentiating natural killer cell and macrophage-mediated tumour lysis (Dinarello, 1989; Platanias and Vogelzang, 1990). TNFα and IL-1β are cytokines with overlapping biological properties which form a complex network of interactive signals (Le and Vilcek, 1987) and are produced mainly by cells of the monocytes–macrophage series (Nathan, 1987). It has been reported that TNFα is produced from RCC cells (Gogusev *et al*, 1993), but Waase *et al* (1992) reported that mRNA of TNF was expressed in infiltrating monocytes–macrophages in RCC but never by the tumour cells, and that the degree of TNF-α production in RCC depends on tumour spread to the draining lymph nodes. In our study, when the levels in the culture supernatant of short-term and long-term established cells were measured, TNFα and IL-1β levels were lower than the minimum detectable concentrations in many cases, suggesting that they are mainly produced by monocytes–macrophages. There are very few studies on serum TNFα and IL-1β in RCC patients. Bichler *et al* (1990) reported that when serum TNFα level was measured in 29 RCC patients, patients with metastatic disease could exhibit increased levels of serum TNF and that the decrease in TNF may be associated with a better prognosis.

Dosquet *et al* (1997) reported that plasma TNFα level was higher in RCC patients compared to healthy controls, and that it was higher in the disseminated group compared to the undisseminated group. They also reported that in multivariate analysis for proportional hazard regression, only TNFα was an independent prognostic indicator, with a normal plasma TNFα being highly predictive for a good prognosis in patients with RCC (Dosquet *et al*, 1997). Hamao *et al* (1994) reported that serum TNFα was elevated in 6 out of 32 patients (18.8%) and serum IL-1 was elevated in 1 out of 32 patients (3.1%). They also reported that the number of serum TNFα-positive patients increased with stage. In this study, we were able to measure serum TNFα and serum IL-1β levels in all the control subjects and RCC patients by using a highly sensitive ELISA kit. Serum TNFα level was significantly higher in the stage I, II, III and IV groups compared to the control group, and unlike serum IL-6, it was already increased in the low stage. There was no significant difference in serum IL-1β level among the groups. As for grade, it was higher in the grade 1, 2 and 3 groups compared to the control group, and unlike serum IL-6, it was already increased in grade 1. In the ROC curve analysis, AUC of serum TNFα was best compared to other cytokines. These results indicate that the production of IL-6 in RCC patients is related to parameters of tumour progression such as tumour size and the existence of metastasis. As for TNFα, it was already higher in the stage I group and grade 1 group compared to the control group, so that it may be useful in early diagnosis and post-operative follow-up. TNFα has been directly shown to serve as anticancer bioactive molecules. In addition, TNFα indirectly exerts antitumour effects by activation of T cells, NK cells and macrophages. The increased TNFα secretion in RCC patients may be considered as a favourable compensatory mechanism to counteract tumour growth. However, TNFα mediates the catabolism of the body substrate. The increased TNFα secretion may induce a hypermetabolic status that may be a cause of malnutrition and cancer cachexia. Further studies on the role of these cytokines in tumour progression should provide some insight into RCC treatment.
